# Third meeting of the Australian and New Zealand Purine Club

**DOI:** 10.1007/s11302-023-09961-y

**Published:** 2023-08-18

**Authors:** Reece A. Sophocleous, Ronald Sluyter, Carolina Gubert, Srdjan M. Vlajkovic, Jennie M. E. Cederholm

**Affiliations:** 1 Molecular Horizons and School of Chemistry and Molecular Bioscience, University of Wollongong, Wollongong, NSW 2522 Australia; 2 The Florey Institute, The University of Melbourne, 30 Royal Parade, Parkville, VIC 3052 Australia; 3 Department of Physiology and Eisdell Moore Centre, Faculty of Medical and Health Sciences, The University of Auckland, Auckland, 1142 New Zealand; 4 Translational Neuroscience Facility and Department of Physiology, School of Biomedical Sciences, UNSW Sydney, Sydney, NSW 2052 Australia

**Keywords:** Adenosine receptor, P1 receptor, P2X receptor, P2Y receptor, Purinergic signalling

Purinergic signalling research has steadily expanded globally since the late Professor Geoffrey Burnstock proposed ATP as a neurotransmitter and established purinergic signalling as a novel concept in 1972 [1]. Parallel to the expansion of purinergic signalling research, Purine Clubs have been founded in several countries across the world to bring together scientists interested in this field, with the first formalised in Italy in 1991 [2]. The Australian and New Zealand (ANZ) Purine Club is relatively new, having been initiated and formed in 2018 with Prof. Burnstock (Past President), Dr. Jennie Cederholm and Prof. Ronald Sluyter (Co-Presidents), and Assoc. Prof. Srdjan Vlajkovic (New Zealand representative) comprising the founding Executive Committee [3]. The Executive Committee expanded in 2020 when Dr. Carolina Gubert (Victorian representative) and Dr. Reece Sophocleous (Early-career Research representative) joined. In the 5 years of the ANZ Purine Club, three meetings have been held, with the inaugural meeting taking place in Melbourne, Australia, in May 2019 [3]. The second meeting followed in July 2021 and was held virtually due to COVID-19 pandemic travel restrictions [4]. We were delighted to meet up in person for the third meeting of the ANZ Purine Club on May 15, 2023, in the School of Biomedical Sciences, The University of New South Wales (UNSW) Sydney (Sydney, Australia). The Executive Committee organised a programme comprising four sessions including a virtual presentation from invited speaker Prof. Alexei Verkhratsky (University of Manchester, UK). The programme also included the Burnstock Oration, awarded to an ANZ Purine Club member who has made significant contributions to purinergic research regionally and internationally. The recipient of the 2023 Burnstock Oration was Prof. Peter Thorne (University of Auckland, New Zealand). Prof. Thorne completed a PhD at the University of Auckland, followed by post-doctoral studies at the Kresge Hearing Research Institute, University of Michigan. Prof. Thorne is a leading auditory neuroscientist with research interests focussed on mechanisms, diagnosis, and treatment of inner ear diseases and clinical and population health approaches to prevention and alleviation of hearing loss. The research conducted by Prof. Thorne has demonstrated the role of purinergic signalling in cochlear development, maintenance of electrochemical homeostasis, auditory neurotransmission, and response to stress [5]. Prof. Thorne’s work has been cited over 5000 times. Furthermore, he has held senior management positions at the University of Auckland, where he led the establishment of the Section of Audiology and the Master of Audiology degree programme. He is currently the Director of the Eisdell Moore Centre and co-lead of the Aotearoa Brain Project. Prof. Thorne also contributes substantially to the hearing-impaired community, serving as the President of the National Foundation for the Deaf, and was one of the lead advocates for the establishment of Newborn Hearing Screening through Project Hearing Impairment and Early Detection and Intervention (HIEDI). In 2009, he was made a Companion of the New Zealand Order of Merit (CNZM) for services to audiology and auditory neuroscience. Prof. Thorne’s presentation at the meeting highlighted the role of purinergic signalling in the cochlea and particularly the role of P2X receptors in adaptation to noise.

The meeting also paid tribute to the late Prof. James (Jim) Wiley (1936–2022), who passed away following a short battle with renal cancer on 28th December 2022. Prof. Wiley pioneered purinergic research in Australia and abroad [6]. Long-term collaborator, mentee, and former student Dr. Ben Gu (The Florey Institute, Australia) presented an overview of Prof. Wiley’s contributions to the field of purinergic signalling, especially P2X7 receptor signalling. This presentation included his early studies on the P2X7 receptor in chronic lymphocytic leukaemia lymphocytes, including the discovery that activation of the P2X7 receptor mediates the rapid shedding of L-selectin (CD62L) from these cells and that the P2X7 receptor is inhibited by KN-62, commonly used to study P2X7 receptor signalling. Dr. Gu also gave a detailed account of the discovery and identification of five single-nucleotide polymorphisms in the *P2RX7* gene, which encode either a loss or gain of P2X7 receptor activity and the discovery of the P2X7 receptor as a scavenger receptor with a role in innate immunity (phagocytosis). Furthermore, Dr. Gu outlined other seminal findings from his collaborations with Prof. Wiley, including the role of different metalloproteases in the P2X7 receptor-mediated shedding of L-selectin and the low-affinity IgE receptor (CD23), and the roles of P2X7 receptor activation in matrix metalloproteinase-9 release from leukocytes, apoptosis of platelets, and membrane fluidity.

Prof. Alexei Verkhratsky presented a historical overview of purinergic signalling in the nervous system, dating from the evidence of purines in early evolution up to the birth of purinergic neurotransmission as a concept in 1972. Prof. Verkhratsky covered studies of his own and those of others, highlighting the development of our understanding of ATP as a neurotransmitter, amongst its other cellular roles.

The conference included six presentations by higher degree research students. Yihan Li (The Florey Institute) won the Best Student Presentation Award for presenting data based on their recently published article [7]. Yihan revealed that the expression of cell-surface P2X7 receptors is decreased on peripheral blood leukocytes in subjects positive for beta-amyloid plaques with either cognitive impairment or dementia, providing new insights into the role of P2X7 receptors in Alzheimer’s disease and its potential as a biomarker in this disorder. Bill Chan (University of Sydney, Australia) presented work in collaboration with Vast BioScience (Queensland, Australia), which utilised fragment-based drug discovery in conjunction with homology modelling using the zebrafish P2X4 receptor to develop novel, non-planar P2X4 receptor antagonists. Chiara Dane (University of Sydney) provided an overview of ongoing work on the production and elucidation of key pharmacophoric features of newly designed analogues of selective P2X4 receptor antagonists, for screening as inhibitors of microglial activation. Rounding off a series of presentations on purinergic receptors and drug discovery, Ben Ma presented on the design and synthesis of fluorinated ligands of the P2Y_12_ receptor, highlighting strategies and challenges of targeting the P2Y_12_ receptor in the central nervous system [8].

Julia-Ciara Schnell (University of Wollongong, Australia) presented evidence that the P2X4 receptor, and to a lesser extent the P2X7 receptor, contributes to the mitogen-induced proliferation of human T cell subsets and that gene transcripts for these receptors are expressed in the spleen, liver, and lungs of humanised mice with graft-versus-host disease, a common complication following donor haematopoietic stem cell transplantation. Extending findings from her previous presentation at the second meeting of the ANZ Purine Club [4], Amal Elhage (University of Wollongong) revealed that an anti-human P2X7 receptor monoclonal antibody (clone L4) [9] reduces clinical and histological graft-versus-host disease in humanised mice and that this effect is associated with an increase in human regulatory T cells and a decrease in human T helper 17 cells. Post-doctoral researcher Dr. Prathamesh T Nadar Ponniah (UNSW Sydney) presented novel data on purinergic hearing adaptation (PHA), an otoprotective mechanism involving P2X2 receptors. This data suggested that PHA has similar kinetics at normative physiological sound levels (71-75 dB SPL), as previously shown in loud noise (85 dB SPL) [10], suggesting that P2X2 receptor-mediated cochlear hearing adaptation is fundamental to the regulation of hearing sensitivity within normal physiological soundscapes to protect hearing function. In further support of early-career researcher development, an ongoing goal of the ANZ Purine Club, sessions were chaired by early-career researchers Tahnee McEwan (University of Wollongong), Dr. Fayaz Ali (University of Sydney), and Dr. Reece Sophocleous (University of Wollongong).

In summary, this third meeting of the ANZ Purine Club was an excellent opportunity to meet and discuss the latest purinergic signalling research in the Asia-Pacific region. It provided and will continue to provide an important forum for the Club’s early-career researchers to network and establish connections with more senior members. With the ANZ Purine Club recently having changed Executive Committee membership during the Annual General Meeting in July 2023, with Assoc. Prof. Stephen Fuller (University of Sydney) appointed as the new Club President, we are excited to see the Club continue to prosper under the new leadership and look forward to the Club’s further growth, new collaborations, and next meetings in the coming years.

## Data Availability

Not applicable.
